# A comparative analysis of gamma and neutron radiation shielding properties of Gd_2_O_3_ nanoparticles within HDPE composites irradiated with argon ion beam

**DOI:** 10.1038/s41598-026-40153-x

**Published:** 2026-03-11

**Authors:** Mohamed Shabib, Eman. K. Tawfik, A. M. Abdel Reheem, Afaf Nada, H. A. Ashry

**Affiliations:** 1 Radiation Physics Department, National Center for Radiation Research and Technology (NCRRT), Egyptian Atomic Energy Authority, P.N, Cairo, 11787 Egypt; 2 Physics Department, Faculty of Women for Art, Science and Education, Ain Shams University, 1, Asma Fahmi Street, Heliopolis, Cairo, 11757 Egypt

**Keywords:** Gamma and neutron shielding, Gadolinium oxide (Gd_2_O_3_) nanoparticles, Argon ion irradiation, High-density polyethylene (HDPE), Materials science, Nanoscience and technology, Physics

## Abstract

Gd_2_O_3_/HDPE nanocomposite materials were prepared using the sol-gel method. These composites were developed to investigate the gamma-ray and neutron shielding properties of HDPE reinforced with Gd_2_O_3_ nanoparticles at different concentrations (x = 4.0%, 12.0%, 20.0%, 30.0%, and 40%). The study also investigates the effects of argon ion irradiation on the gamma-ray and neutron shielding properties. The composite was irradiated with argon ion beam of energy 4 keV, to a fluence of 22 × 10^16^ ions/cm^2^. Different analytical techniques were applied to study the Gd_2_O_3_/HDPE nanocomposites. The mass attenuation coefficient (µ_m_) was experimentally measured using the Eu-152 gamma point source. The HPGe detector was used for measurement of shielding parameters of unirradiated and irradiated composites. The (µ_m_) was measured at different photon energies, and the outcomes have been contrasted with those obtained utilizing the NIST-XCOM software. There was an acceptable agreement between the theoretical and experimental results. For example, at 121 keV, the attenuation parameter increases from 0.1845 for pure HDPE to 0.5065 for the 30 wt% Gd_2_O_3_/HDPE composite, corresponding to an enhancement of approximately 175%. Also, total neutron macroscopic cross-sections were evaluated for both irradiated and unirradiated samples. Results revealed a significant enhancement in gamma and neutron attenuation post-irradiation, attributed to structural, mechanical, and morphological changes induced by the ion beam. For pure HDPE, Ʃ_T_ increases by 33%, whereas the 12 wt% and 20 wt% Gd_2_O_3_/HDPE composites exhibit further enhancements of approximately 82% and 70%, respectively. The findings indicate ion treatment provides a promising method for improving radiation shielding parameters of polymer nanocomposites.

## Introduction

Research into radiation shielding materials has advanced significantly to address the growing use of radiation-based technologies. Rising global energy demands are driving the development of modern nuclear power plants, which increases the need to prevent environmental radioactive contamination. This underscores the urgency of creating effective shielding solutions to protect ecosystems and human health. Consequently, the advancement of radiation-attenuating materials has emerged as a critical scientific priority, given their indispensable role in ensuring biosafety. High-energy ionizing radiation, such as neutron emissions and gamma rays, poses serious risks to human health by damaging cells and DNA through direct exposure. These phenomena are associated with deterministic tissue complications (e.g., acute radiation dermatitis) and stochastic effects, such as genomic instability, teratogenic mutations, and transgenerational neuropathological sequelae. Experimental investigations have substantiated widespread public worry toward radiation-associated technologies, largely attributable to perceived risks of stochastic radiation exposure events^1–5^. High-performance shielding materials are critical to reduce hazardous radiation exposure and maintain safe conditions in these environments^6^. Radiation shielding applications require materials with optimal density, thermal stability, structural durability, corrosion resistance, and cost-efficiency. Overall, research has studied several shielding radiation materials, including ceramics^7,8^, glass systems, polymer composites^9–11^, and metal alloys^12,13^aimed at developing effective gamma-ray and neutron shielding materials.

While the fundamental concept of neutron shielding is well understood, its implementation is more complex than shielding against gamma radiation due to the need to account for a broad spectrum of neutron energies. The primary goal of neutron shielding materials is to effectively absorb fast, high-energy neutrons, rather than focusing on slower, low-energy thermal neutrons. Neutron shielding often employs hydrogenated materials to slow fast neutrons to thermal energy, which are then absorbed by specific materials. However, shielding high-energy neutrons is complex, as gamma rays from the neutron source (e.g., nuclear reactions) and those resulting from neutron interactions within the shielding material must also be prevented from passing through^14^. Common materials employed to block neutron radiation differ based on the area of application. Concrete is the material most commonly used for neutron shielding in radiation protection reactors and facilities. Still, cracks can appear in concrete after extended exposure to radiation and are hard to manage as waste or replace^15,16^. This motivated researchers to synthesize and find other materials to achieve this task^17–21^.

According to earlier research, such as europium, gadolinium, and samarium, that have atomic numbers between 57 and 71 even have the ability to absorb neutrons larger than those of ^10^B. These elements are already employed as neutron-absorbing and reflecting materials^22–25^. Among these elements, Gd has a relatively high atomic number 64. The incorporation of Gd into the matrix significantly improves the gamma-ray shielding performance. Several studies have utilized Gd_2_O_3_ with different matrices as a dual radiation shielding material against gamma rays and neutrons^5,20,26–28^. The ideal material to absorb thermal neutrons is gadolinium, which has thermal neutron absorption cross sections as high as 62,540 b for Gd-155 and 255,000 b for Gd-157^29^or example, Zhang P. developed a Gd-doped aluminum plate that, in terms of neutron shielding, was nearly identical to the 30%wt. B_4_C/6061Al composite and the 10%wt. Gd_2_O_3_/6061Al composite^30^.

Ion irradiation effectively modifies the morphology of thin films, alters surface energy, and displaces atoms, resulting in unique phenomena that enable precise patterning and device fabrication^17^. Using plasma techniques, polymers can be modified at a structural and chemical level, affecting their overall behavior, such as roughness, to enhance adhesion and create well-defined surface nanostructures^31,32^. SRIM/TRIM calculation code^33,34^is utilized to study the behavior of ion beams in target materials. It examines various factors, such as the total displacement of target atoms, the number of vacancies, and the occurrence of replacement collisions. This code is also employed to evaluate the damage caused by energetic ions, the energy loss of the ions utilized, and the penetration range of the ions within the target material^35,36^. The present study is intended to use Gd_2_O_3_ as dual gamma and neutron shielding to prepare an advanced flexible composite structure for radiation shielding against neutrons and gamma rays using High-Density Polyethylene (HDPE) as a matrix. In addition, the effects of low-energy argon ion irradiation current bombardment on Gd_2_O_3_/HDPE composites are evaluated, which have not been widely reported in previous studies. Mass attenuation coefficients (µ_m_) were measured at wide range photon energies emitted from Eu-152 gamma point source (from 121KeV to 1408KeV), and have been theoretically estimated using the X-COM program for comparison. The neutron macroscopic cross section is also studied.

## Materials and methods

### Materials

All types of chemicals, including gadolinium (III) nitrate (Gd (NO_3_)_3_, HDPE, xylene (C8H10), NH_4_OH, and nitric acid, were obtained from Sigma-Aldrich of Germany and utilized as received without further purification.

### Preparation of gadolinium oxide nanoparticles

Gd_2_O_3_ nanoparticles were synthesized via the sol-gel technique, starting with 4 g of gadolinium nitrate hexahydrate (Gd (NO_3_)_3_·6H_2_O), which was dissolved in 160 mL of deionized water. For one hour, at 90 °C, the solution was heated while being constantly stirred. Following this, NH_4_OH was introduced into the mixture. The resulting gel was subsequently washed multiple times using deionized water and ethanol, then, for 5 min, centrifuged at 3500 rpm to isolate the product. The gel was then dried in an oven at 100 °C for 12 h. Finally, the calcination process was conducted at 800 °C for 5 h.

### Preparation of HDPE/Gd_2_O_3_ nanocomposites

Seven grams of high-density polyethylene (HDPE) pellets were solubilized in 100 mL of xylene at 135 °C under constant stirring for three hours. The predetermined quantities of Gd_2_O_3_ nanoparticles (4%, 12%, 20%, 30%, and 40% by weight) were subjected to ultrasonic treatment to disperse the material in xylene for 30 min. The prepared Gd_2_O_3_ suspension was added to the pure HDPE solution and subjected to reflux at 140 °C for one hour under vigorous stirring to form a uniform composite. The composite mixtures were cast and subsequently dried in an oven at 50 °C until a constant weight was achieved.

### Characterization

The prepared Gd_2_O_3_/HDPE nanocomposites were characterized using several analytical techniques, including SEM, XRD, FTIR, EDX, and tensile testing for mechanical properties. XRD was employed to explore the crystalline structure of the synthesized Gd_2_O_3_ and Gd_2_O_3_/HDPE nanocomposites using copper (Cu) Kα radiation with a wavelength of 1.5406 Å, scanned at a rate of 8° per minute across a 2θ range from 5° to 80°. SEM was utilized to examine surface morphology and elemental distribution, with EDX confirming the elemental composition. FTIR was conducted to analyze molecular interactions and functional groups in the nanocomposites a resolution of 4 cm⁻¹ and across a spectral range of 400–4000 cm⁻¹. Thermal stability and decomposition characteristics were assessed via TGA, providing insights into weight loss behavior with temperature variations. Additionally, mechanical properties were evaluated utilizing a tensile testing equipment (Dongguan Haida Equipment Co., Ltd., China; Qchida computerized testing equipment) on dumbbell-shaped Gd_2_O_3_/HDPE nanocomposite samples at 25 ± 2 °C at a crosshead speed of 300 mm/min. The average mechanical measurements were determined from at least three samples.

### SRIM/TRIM calculation

**Fig. 1:**
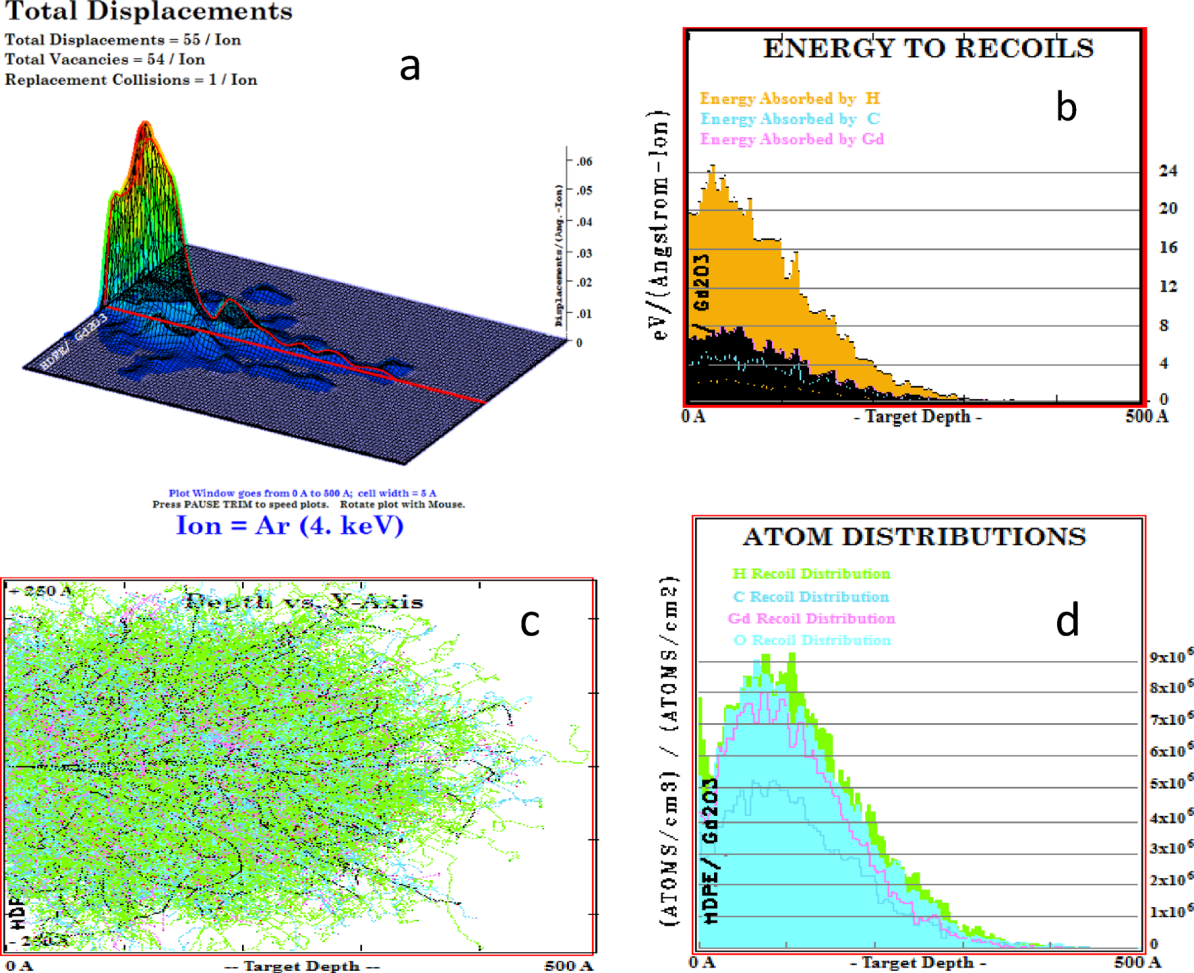
TRIM simulation results for Gd_2_O_3_/HDPE nanocomposites irradiated with 4 keV argon ion **a**) 3D ion distribution image. **b**) The energy from ion to recoils atoms, and **c**) ion penetration depth in target. **d**) The atom recoil distributions.

When an energetic ion collides with a polymer, its penetration depth is determined by both its energy and the target material’s composition. The energy of the incoming ions is lost through both elastic and inelastic collisions between target atoms and electrons. When an ion beam hits the target material, the ions penetrated into it, this process depends on the target nature. Incident ions lose their energy to the target material in various ways, such as displacement, replacement of target atoms, and phonons. The simulation was carried out using the SRIM/TRIM code^33,34^. Figures (1a) shows the total displacements where the displacements atoms are 55 per ion and the target vacancies are 54 per ion also the replacement target atoms are one per ion. An ion dose of 22 × 10^16^ ion/cm^2^ makes 12 × 10^18^ stable vacancies in 1cm^3^. Figures (1b) shows the energy from ion to recoil target atoms; here the energy absorbed by hydrogen atom is larger than the carbon or Gd atoms. Where the total energy from the incident ion is 3.12 keV. The absorbed energy in H, C, Gd, and O are 305,772, 918, and 1190 eV, respectively. Figures (1c) shows projected area for 4 keV argon ion is 40 nm and ion straggling are 33 nm. Also, Figure (1d) demonstrates the loss of energy to phonons, which are the energy contained in atomic vibrations within the target crystal. Every phonon originates equally from recoil atoms and ions.

### Ion irradiation of Gd_2_O_3_/HDPE nanocomposites

**Fig. 2:**
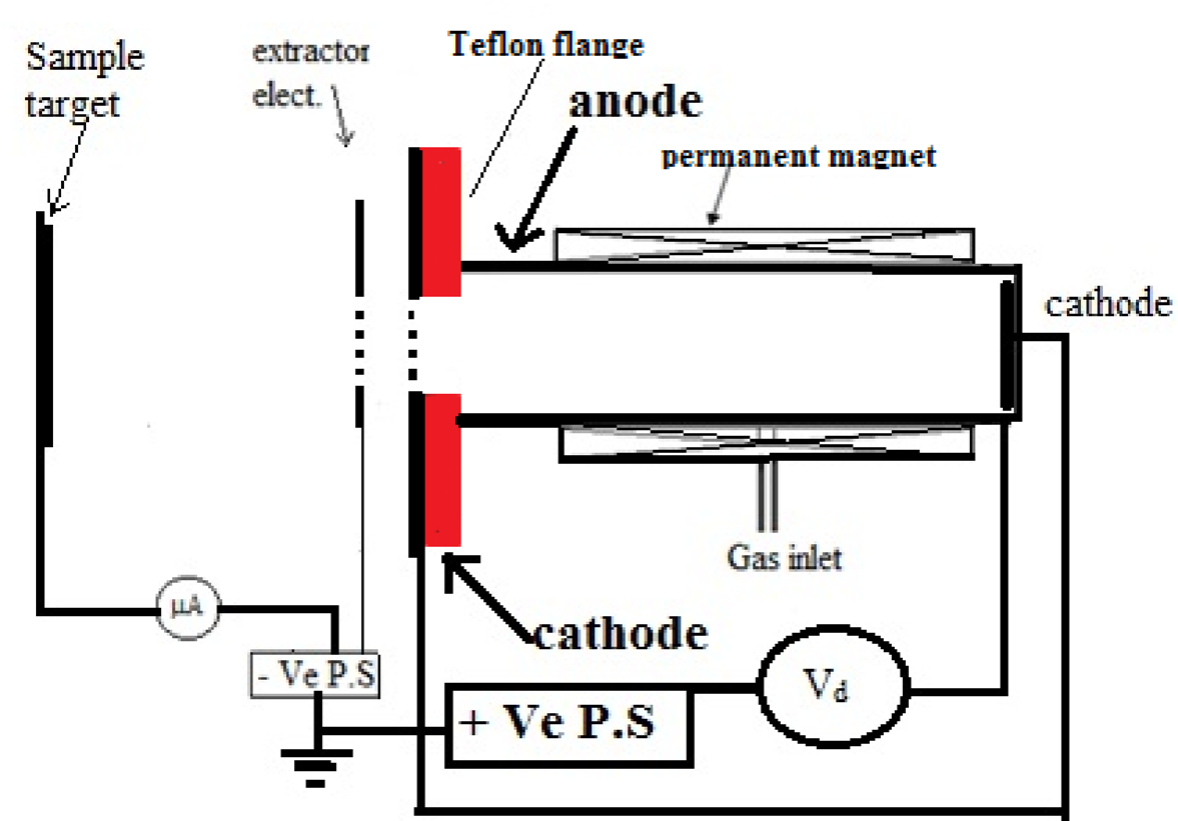
Schematic diagram of cold cathode penning ion source.

Figure 2 presents a schematic describing the configuration of the cold grid cathode Penning ion source utilized for samples irradiation^37^. It includes a stainless-steel anode cylinder with a diameter of 30 mm and a length of 60 mm, as well as two stainless-steel disc cathodes each measuring 50 mm in outer diameter. One cathode’s inner surface has an 18 mm diameter semi-transmittance. An 8 mm thick Teflon insulator flange separated the anode and cathode. The collector (sample target) plate is placed 50 mm from the extraction electrode to measure the argon ion beam output current. The anode is surrounded by a magnetic field to condiment the plasma inside the cylinder. The samples are irradiated using argon fluence equals 22 × 10^16^ ion.cm^−2^ with energy of 4 keV. The operational pressure was maintained at 1.2 × 10⁻³ mbar, the current density was 120µA/cm^2^, and the discharge current (I_d_) was 3 mA.

#### Gamma-ray shielding properties

The gamma-ray shielding performance of the samples was assessed using a well-defined experimental setup, as illustrated in our previous study^38^. The investigation utilized photon energies ranging from 121 keV to 1408 keV emitted from an Eu-152 point source. The gamma-ray energy peaks were detected using a high-purity germanium (HPGe) detector with a relative efficiency of 103.5% at 1.33 MeV for Co-60, which has a relative efficiency of 103.5% for Co-60 at 1.33 MeV, and features dimensions of 81.6 mm in diameter and 99.5 mm in length. Gamma Vision software was used to analyze the γ-ray spectra of both the unattenuated beam and the beam transmitted through the nanocomposite samples, enabling determination of the count rate for each characteristic energy peak. All measurements were acquired over a duration of one hour. For each sample, the experiment was repeated three times, and the mean value was taken as the final result. Background radiation was measured separately and subtracted from all recorded spectra.

The linear attenuation coefficient (µ) characterizes the overall probability of interaction. Equation (1) provided the linear attenuation coefficients (µ) if the intensity of the γ-ray through every sample (I) and the preliminary intensity of the γ-ray energy (Io) were measured:

1 $$\:\mu\:=-\frac{1}{x}In\frac{I}{Io}$$

(1)

The variable *x* denotes the thickness of the sample under investigation. The µ values corresponding to specific gamma energies were calculated by evaluating the slope of the logarithmic ratio between transmitted and incident intensities. The mass attenuation coefficient (µₘ) can be calculated by dividing the linear attenuation coefficient (µ) by the sample’s density (ρ, in g·cm⁻³).

To assess the experimental uncertainty/error in the µ_m_, the expression given by Eq. (2) was applied^39,40^. This uncertainty results from variations in three key factors: the sample thickness (x), and an estimate of the peak area represented by Io and I.

2 $$\:{\varDelta\:\mu\:}_{m}=\frac{1}{x}\sqrt{{\left(\frac{\varDelta\:{I}_{o}}{{I}_{o}}\right)}^{2}+{\left(\frac{\varDelta\:I}{I}\right)}^{2}+{\left(\frac{\varDelta\:\mathrm{x}}{\mathrm{x}}\right)}^{2}}$$

Here, $\:\varDelta\:{I}_{o}$, $\:\varDelta\:I$, and $\:\varDelta\:\mathrm{x}$ represent the error of measurements for I₀, I, and x in every sample, respectively. It was estimated that the results’ statistical error was less than 2%.

The theoretical mass attenuation coefficients (µₘ) are provided by the Hubbell and Seltzer database. Utilizing input parameters, Berger and Hubbell’s XCOM software calculates µₘ values, which represent photon interaction cross sections for a wide range of materials such as elements, compounds, and mixtures over an energy range spanning from 1 keV to 100 GeV^41^. Recently, various researchers have used this software to establish the µ_m_ of radiation shielding materials theoretically^42–44^.

### Neutron shielding characteristics

Neutron shielding parameters were estimated by determining the ratio of transmitted and incident neutron count rates using an ^241^Am-Be neutron source with activity 37 GBq (1 Ci). The experimental setup in details was illustrated in our previous study^38^. All required measurements were recorded over a period of 30 min. For each specified sample, the experiments were repeated four to five times to reduce random fluctuations and improve data precision. The following relationship was used to calculate the total neutron macroscopic cross-section:

3 $$\:{\varSigma\:}_{T}=-\frac{1}{x}In\frac{I}{Io}\:\:\:\:\:\:\:\:\:\:$$

Where Σ_T_, x, Io, and I represent the total neutron macroscopic cross section, sample thickness, the initial intensity, and the final intensity, respectively. The parameters in Eq. (2) were applied to calculate the statistical errors for neutron shielding experiments. The results showed an estimated statistical error of less than 5%.

## Results and discussion

### XRD characterization

**Fig. 3:**
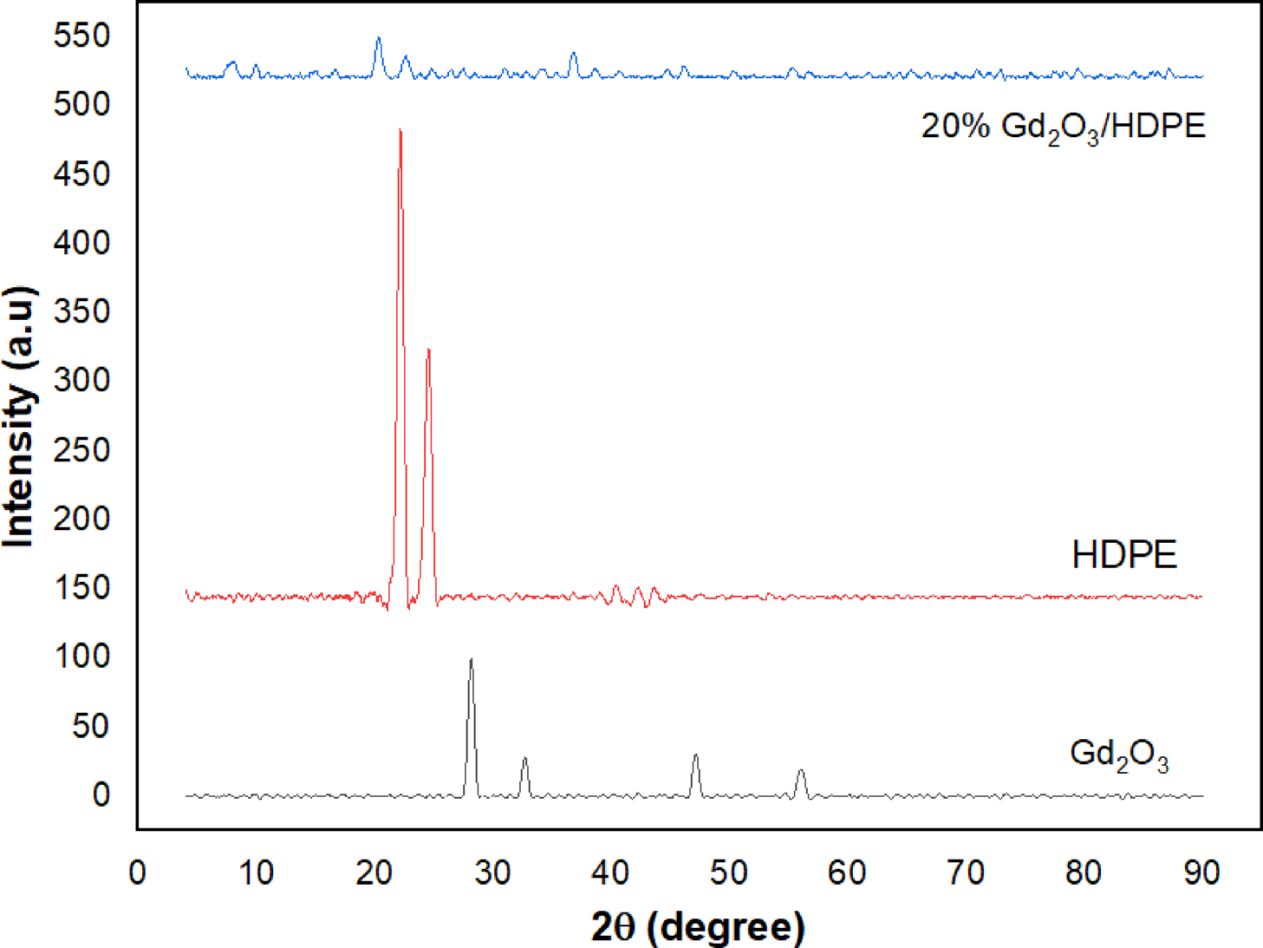
The XRD spectrum of prepared Gd_2_O_3_-NPs, pure HDPE, and 20.0 wt% Gd_2_O_3_/HDPE composite.

XRD provides an efficient and dependable technique for studying the crystalline structure of target materials. This technique highlights different material characteristics, including phase composition, amorphous content, degree of crystallinity, and sample uniformity. The XRD spectra of the produced Gd_2_O_3_-NPs annealed at 800 °C, HDPE, and 20.0 weight% Gd_2_O_3_/HDPE composite are shown in Fig. 3. The analysis verifies the particle size and crystallinity of Gd_2_O_3_ nanoparticles. Distinct peaks observed at 2θ values of 28.14°, 32.62°, and 47.05° align with the reference pattern of α-Gd_2_O_3_ (JCPDS No: 86–2477). Also, lattice parameters demonstrate a crystal structure that is monoclinic^45^. The Gd_2_O_3_ particle size was estimated through the line broadening analysis of X-ray diffraction patterns^46^utilizing the Scherrer Eq. (5) as shown in Table 1.

4 $$\:{\mathrm{D}}_{\begin{array}{c}hkl\\\:\:\end{array}}=\:\frac{\mathrm{K}{\uplambda\:}}{{{\upbeta\:}}_{\mathrm{h}\mathrm{k}\mathrm{l}}\:\mathrm{c}\mathrm{o}\mathrm{s}{\uptheta\:}}$$

5 $$\:{{\upbeta\:}}_{\mathrm{h}\mathrm{k}\mathrm{l}}=\:\frac{2{\uptheta\:}{\Delta\:}\mathrm{x}{\uppi\:}}{180}$$

where D is the particle size in the nanomaterial, k is a constant equal to 0.94, θ is the peak position, β_D_ is the peak width at half maximum intensity, and λ is the radiation wavelength (1.54056 A° for Cu Kα radiation). The HDPE seems to show two peaks: a large one at 22.1° and a less pronounced one at 24.5°. These peaks are caused by the orthorhombic unit cell of HDPE is typically characterized by its (110) and (200) diffraction planes. The outcomes validate the previous papers^47,48^. Between 40° and 60°, many weak peaks are also visible^49^. The incorporation of gadolinium into HDPE results in a slight shift and reduction in intensity of the primary peaks (110) and (200). However, it does not affect the original structure of HDPE, likely due to the minimal quantity of gadolinium introduced^50^.

**Fig. 4:**
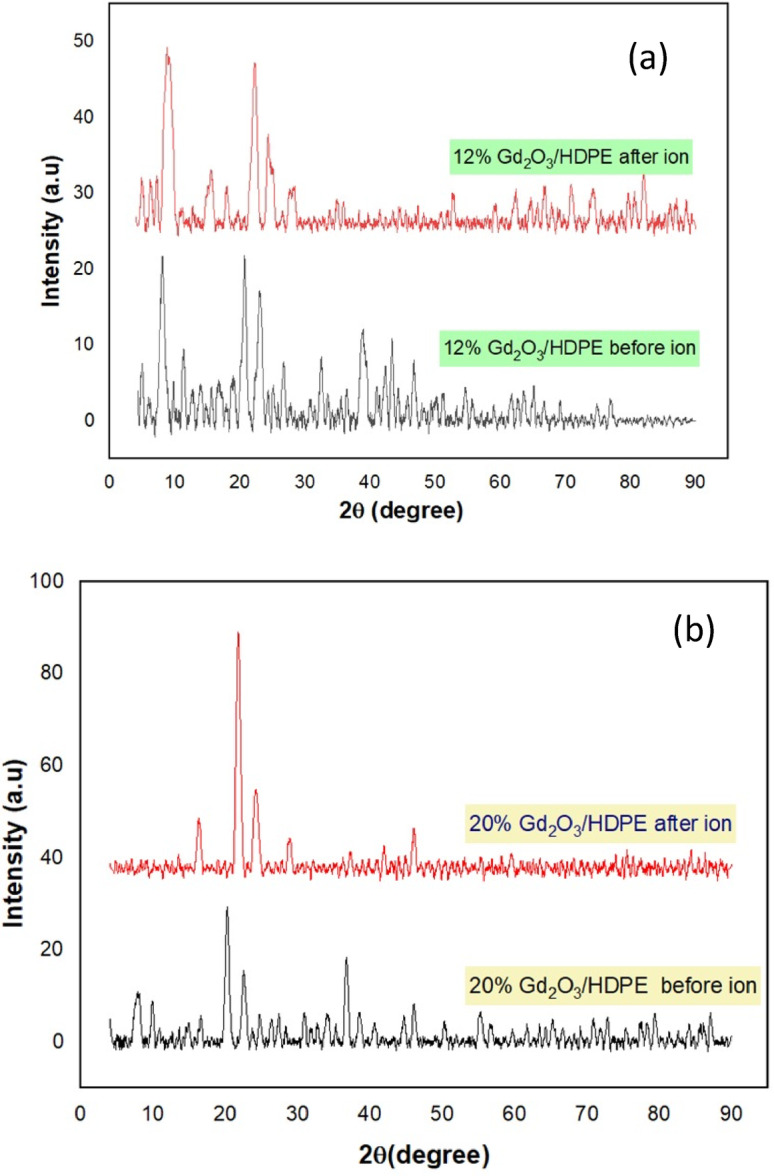
The XRD spectrum of prepared Gd_2_O_3_-NPs: (**a**) 12.0 wt% Gd_2_O_3_/HDPE and (**b**) 20.0 wt% Gd_2_O_3_/HDPE before and after argon ion irradiation.

The ratios 12% and 20.0 wt% Gd_2_O_3_/HDPE have been chosen as representatives to study the effect of argon ion irradiation on the crystalline structure of the prepared samples as shown in Fig. 4. The observed rise in peak intensities may be attributed to argon ion-induced bond scission, which suggests enhanced particle ordering within the polymer films exposed to ion bombardment^51–53^. Since the crystallinity represents a thermodynamically stable phase, scission will be dominant, promoting polymer chain mobility, and facilitating partial molecular recrystallization^54^. The particle size of pure HDPE, pure Gd_2_O_3_, and the ratios of 12% and 20% Gd_2_O_3_/HDPE before and after argon irradiation were calculated using the Scherrer Eq. (5).

**Table 1:** Illustrates the particle size of the HDPE, pure Gd_2_O_3_, 12% Gd_2_O_3_/HDPE and 20% Gd_2_O_3_/HDPE composites before and after ion irradiation to assess their lattice parameters.

Nanomaterial	Peak Position (2θ)	β (FWHM) degree	2ϴ (radian)	K	λ (nm)	Particle Size (nm)
Gd_2_O_3_-NPs	28.14	0.0094	0.2446	0.94	0.154	15.85
HDPE	21.17	0.0170	0.1846	0.94	0.154	8.68
12% Gd_2_O_3_/HDPE before ion	20.53	0.0184	0.1791	0.94	0.154	7.96
12% Gd_2_O_3_/HDPE after ion	22.21	0.0129	0.1937	0.94	0.154	11.43
20% Gd_2_O_3_/HDPE before ion	20.24	0.0107	0.1765	0.94	0.154	13.71
20% Gd_2_O_3_/HDPE after ion	21.73	0.0119	0.1895	0.94	0.154	12.43

The data indicate that argon ion irradiation affects the particle size in Gd_2_O_3_/HDPE nanocomposites. Pure Gd_2_O_3_ has the largest particles (15.85 nm). When added with HDPE, particle size decreases, particularly with 12% loading (7.96 nm), indicating constrained crystal growth. After argon ion irradiation, both 12% and 20% composites show increased particle sizes (11.43 nm and 12.43 nm), likely because of structural reordering. These modifications suggest ion irradiation enhances crystallinity and may enhance the nanocomposites’ efficiency in radiation-exposed environments.

### *FTIR* characterization

**Fig. 5:**
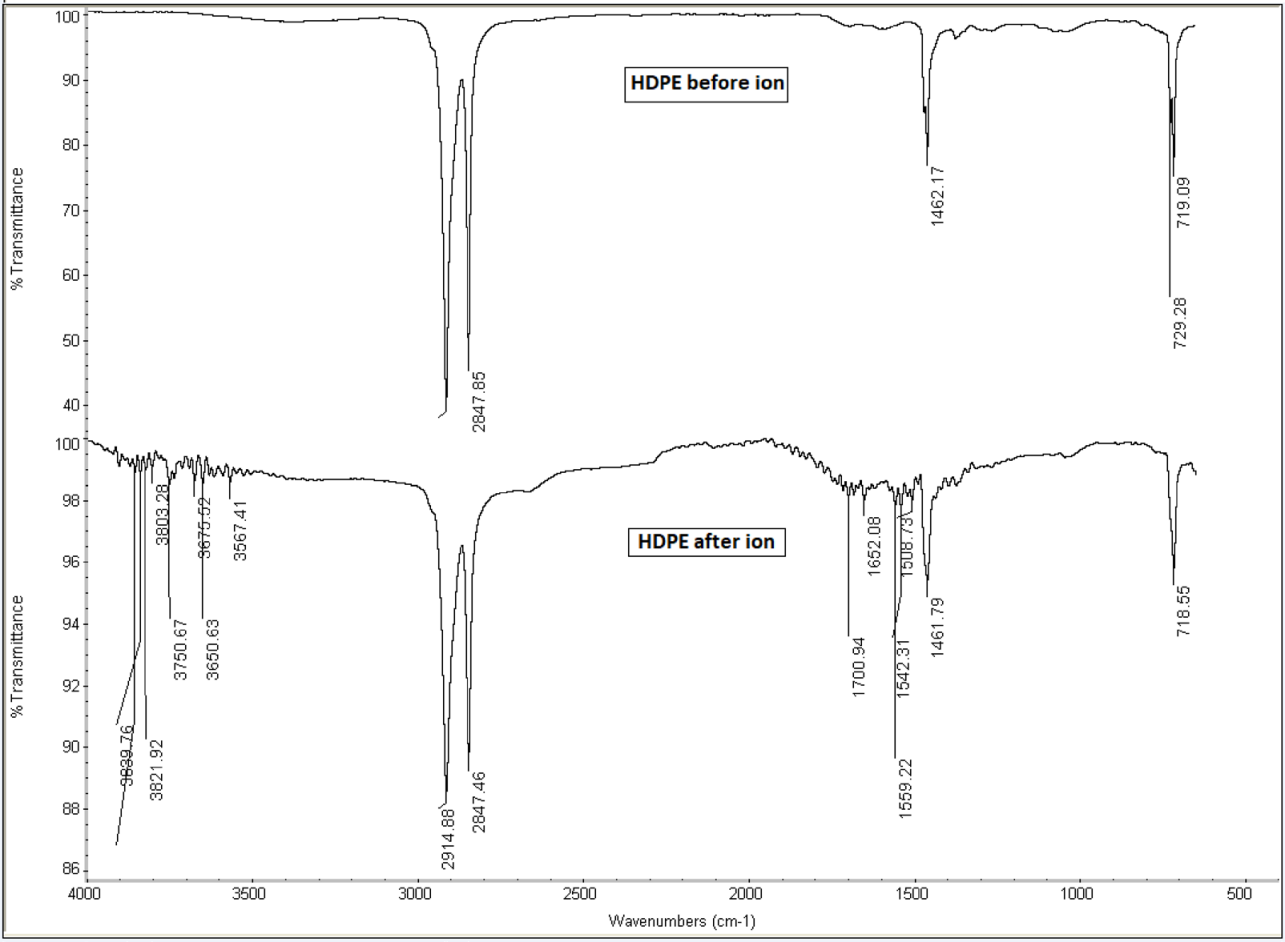
FTIR spectrum of HDPE before and after argon ion irradiation.

Chemical reactions between the HDPE and Gd_2_O_3_ were investigated with FTIR and the region from 4000 to 500 cm^− 1^ was evaluated. FTIR patterns of the HDPE and 20% Gd_2_O_3_/HDPE are shown in Fig. 5. The Functional group absorption bands of HDPE are 2914 Asymmetric CH_2_ stretching, 2847 Symmetric CH_2_ stretching, 1462 CH_2_ scissoring due to bending absorptions of methylene group, and 729 CH_2_ rocking which demonstrates the vibrational mode of the long methylene chain^55,56^. All polymeric samples exhibit stretching vibrations of the methylene group at 2915 and 2840 cm^− 1^, respectively^55^. The sharp band appearing at about 719 cm^− 1^ refers to the rocking vibrations of CH_2_. Owing to the degree of crystallinity in polyethylene, the peak at 719 cm^− 1^ is split, and a further peak is observed around 730 cm^− 1^ in all polymers^57^. The band appearing around 1462 cm^− 1^ refers to the CH_2_ scissors vibrations of all polyethylene^57^.

**Fig. 6:**
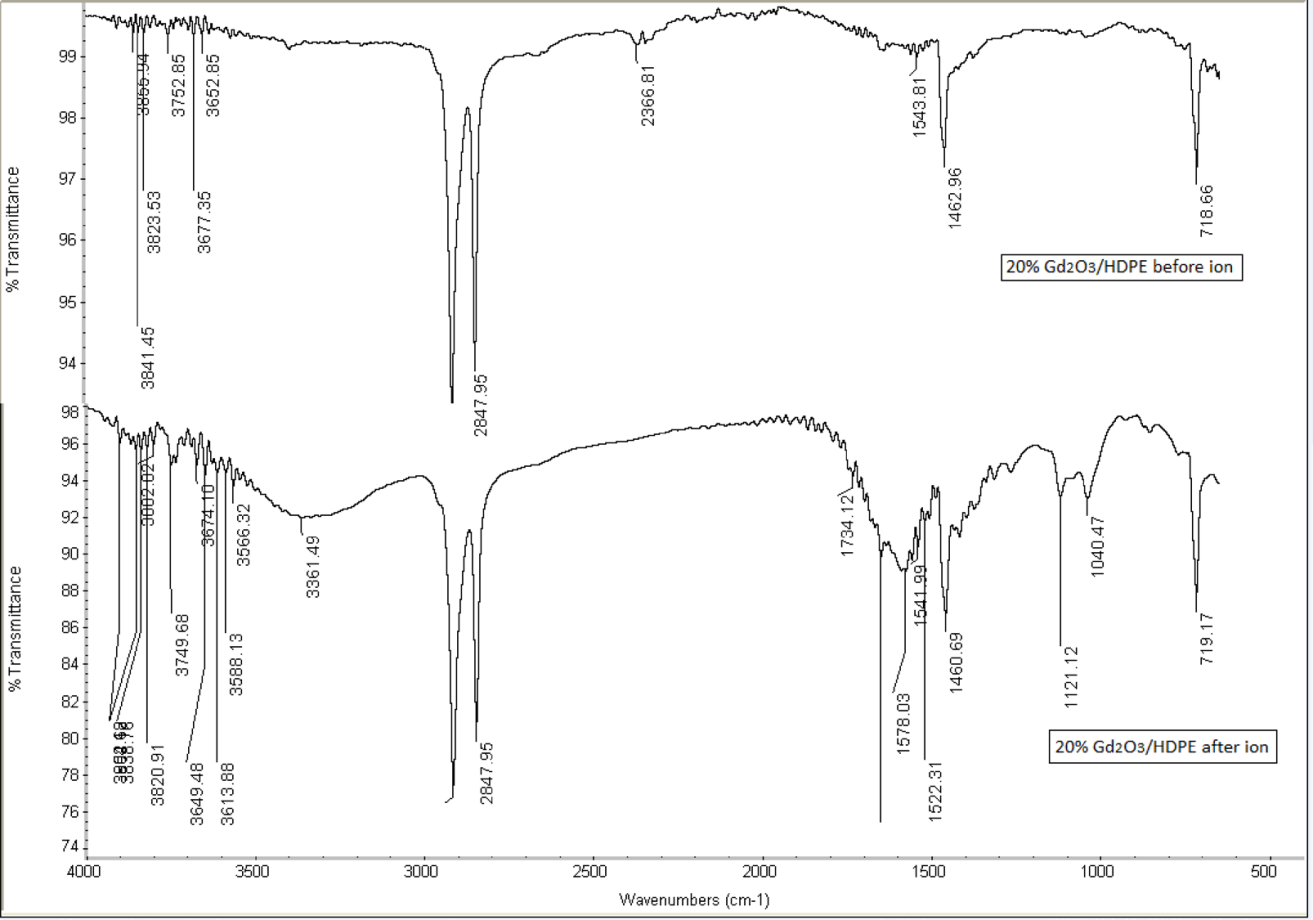
FTIR spectra of pure HDPE and 20.0 wt% Gd_2_O_3_/HDPE before and after irradiation.

In Fig. (6), The bands of Gd_2_O_3_ at 1460 and 2876 cm^− 1^ correspond to bending and symmetric stretching of CH_2_. C-O stretching is represented by the band at 1127 cm^− 1^, while O-H stretching was seen in a broad band between 3100 and 3500 cm^− 1^^58^. The existence of C = O and N–H is indicated by the appearance of weak and broad bands close to 1734 and 3361 cm^− 1^, respectively. This could have been created as a result of the reaction of the polymer when exposed to oxygen and water in the surrounding environment. Moreover, post-irradiation effects may contribute to the formation of these bonds, as similar outcomes have been documented in polymers exposed to heavy ion irradiation^59,60^. The FTIR band range of 3500 cm⁻¹ to 3900 cm⁻¹ is primarily associated with O-H stretching vibrations, particularly those of free (non-hydrogen bonded) hydroxyl groups^61^.

The argon ion irradiation of an HDPE/Gd_2_O_3_ composite leads to the formation of free, non-hydrogen-bonded hydroxyl groups on the surface, evidenced by FTIR bands in the 3500–3900 cm⁻¹ range. This occurs because the energetic argon ions cause bond scission and radical formation in the HDPE, which then react with recoil oxygen atoms as shown in the SRIM simulation. Additionally, the Gd_2_O_3_ nanoparticles undergo sputtering and defect creation, making their surfaces more reactive and contributing to hydroxyl group generation. The sharp, high-wavenumber O-H bands indicate a significant chemical and structural alteration of the composite surface^62^.

### EDX characterization

**Fig. 7:**
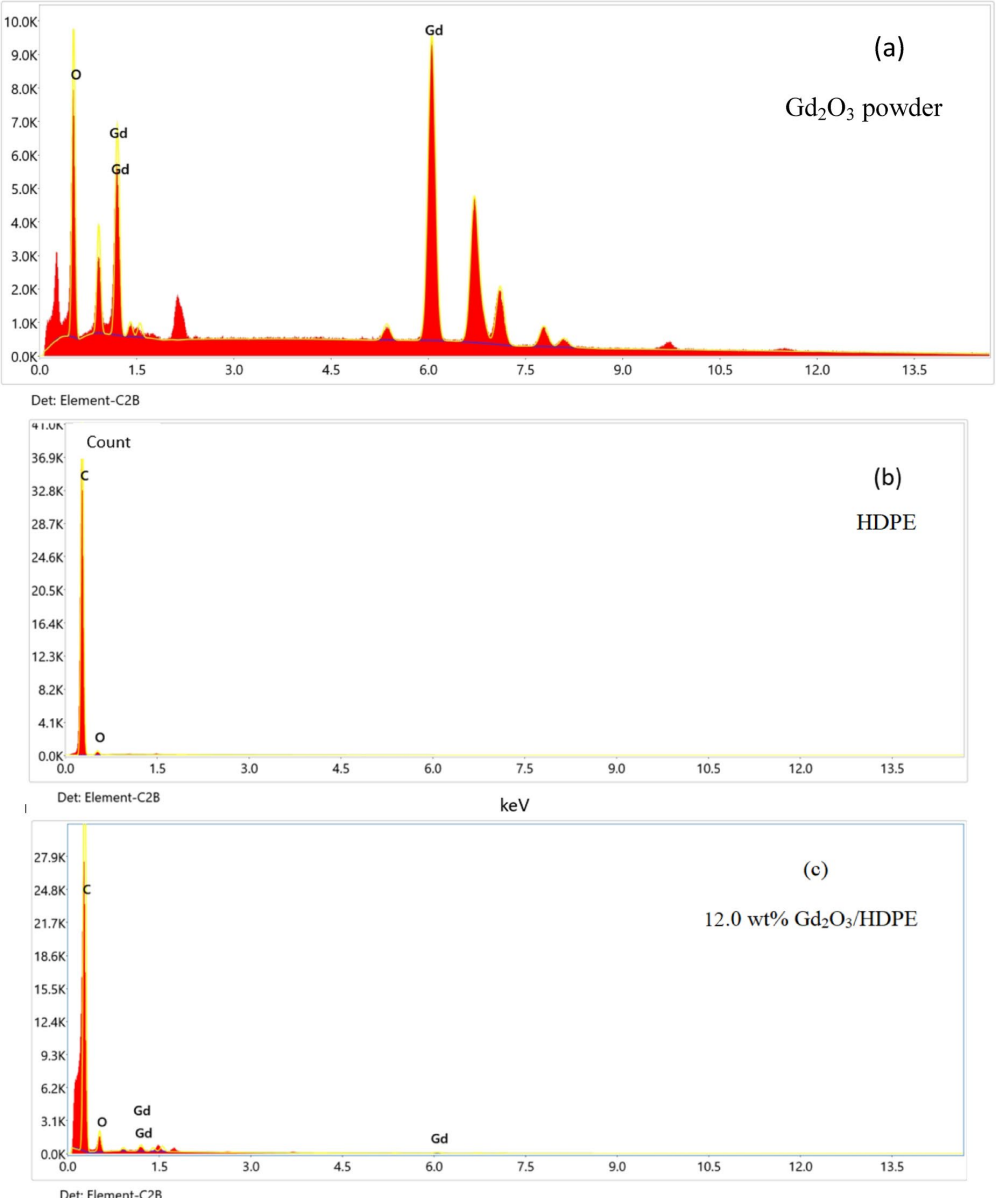

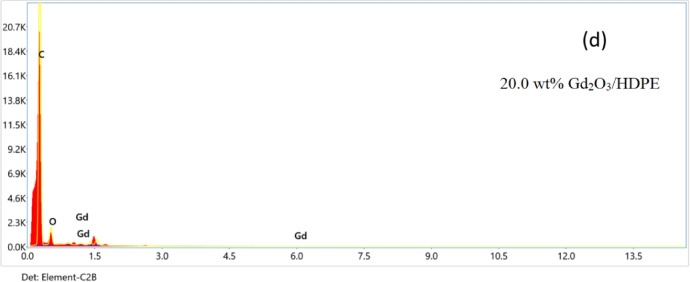
EDX of (**a**) Gd_2_O_3_, (**b**) HDPE, (**c**) 12.0 wt% Gd_2_O_3_/HDPE, and (**d**) 20.0 wt% Gd_2_O_3_/HDPE.

The composition of HDPE pellets, gadolinium powder, and Gd_2_O_3_/HDPE nanocomposites with Gd_2_O_3_ loadings of 12.0 wt% and 20.0 wt% were analyzed using EDX, as shown in Fig. 7. According to Fig. 7c, EDX of the 12.0 weight% Gd_2_O_3_/HDPE sample showed that the elemental compositions were as follows: gadolinium was present at 1.41%, carbon at 90.02%, and oxygen at 8.57%. Further, Fig. 7d shows that the 20.0 weight% Gd_2_O_3_/HDPE sample contained gadolinium at 2.73%, carbon at 92.14%, and oxygen at 5.13%.

### SEM characterization

**Fig. 8:**
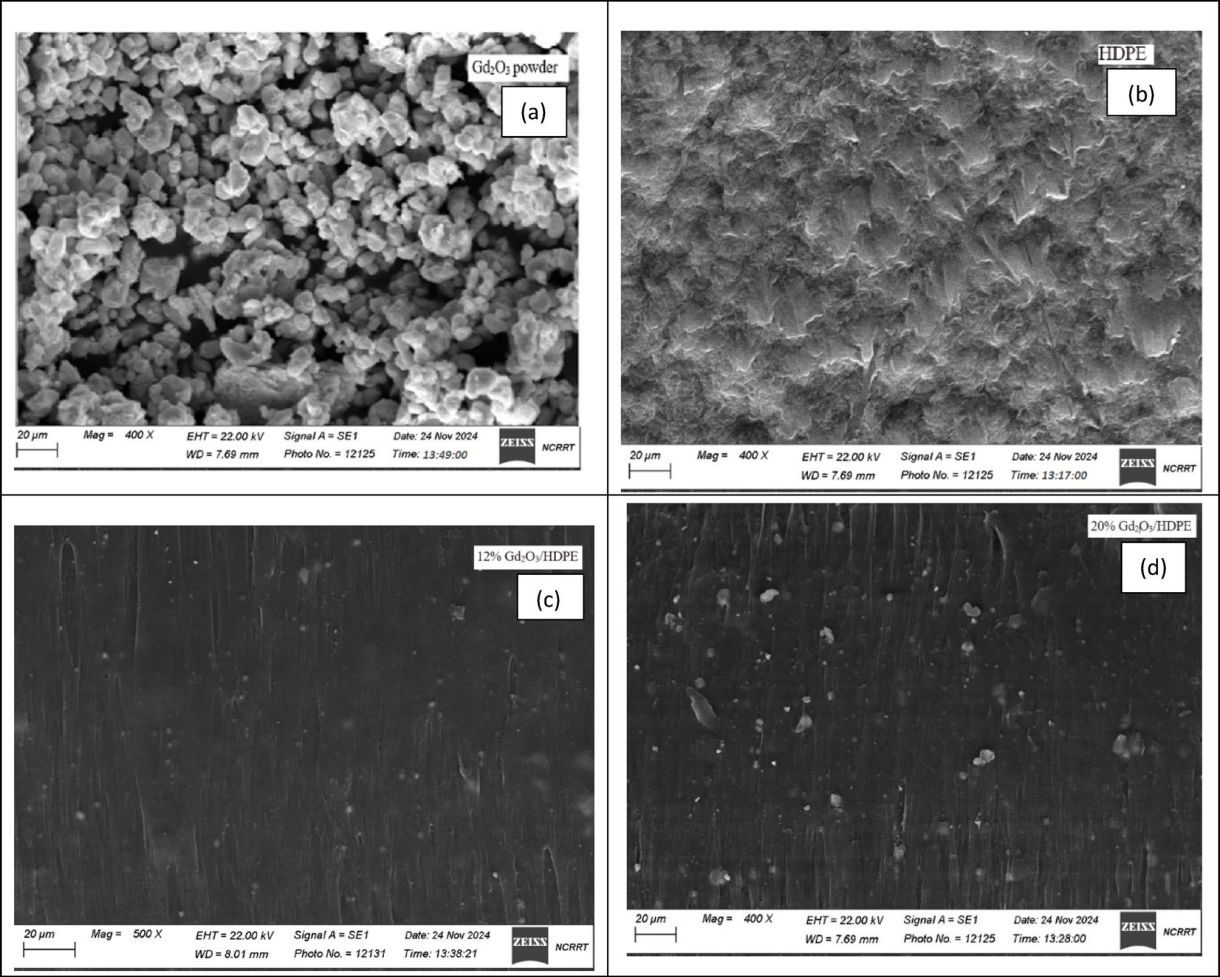
The surface morphology of (**a**) Gd_2_O_3_, (**b**) HDPE, (**c**) (c) 12.0 wt% Gd_2_O_3_/HDPE, and (**d**) 20.0 wt% Gd_2_O_3_/HDPE.

The synthesized samples were investigated using scanning electron microscopy (SEM) to discuss the distribution of gadolinium oxide nanoparticles in the HDPE and variations in surface morphology. Several factors influence the shape of the polymer composite, including processing parameters, melt viscosities of the components, weight fraction, and the compatibility among the composite materials. The dispersion arrangement of the Gd_2_O_3_ filler, HDPE, and HDPE composites containing 12.0 and 20.0 weight% Gd_2_O_3_ nanoparticles are shown in Fig. 8 (a-d). The presence of Gd_2_O_3_ nanoparticles, which mostly have a roughly spherical form, is shown by the finding in figure 6.a. Electron microscopy images of the resulting composites are displayed in Fig. 8 (c-d). Because of the strong interfacial adhesion and good compatibility among the matrix and Gd_2_O_3_ NPs, the figure clearly shows that the Gd_2_O_3_ nano filler is fully immersed in the HDPE matrix and that the HDPE covers the filler. Additionally, it shows that the particle dispersion becomes more uniform when the Gd_2_O_3_ ratio rises from 12.0 wt% to 20.0 wt%, with a noticeable increase in particle density in the HDPE composites corresponds to higher Gd_2_O_3_ concentrations. A more homogeneous distribution of these particles resulted from an increase in the doping ratio of Gd_2_O_3_ NPs. The Gd_2_O_3_ nanoparticles in both composites exhibit uniform dispersion throughout the polymer matrix and homogeneous surface morphologies. Furthermore, no aggregation was observed within the polymer matrix, indicating that the composites were properly formed.

### Mechanical measurements

**Fig. 9:**
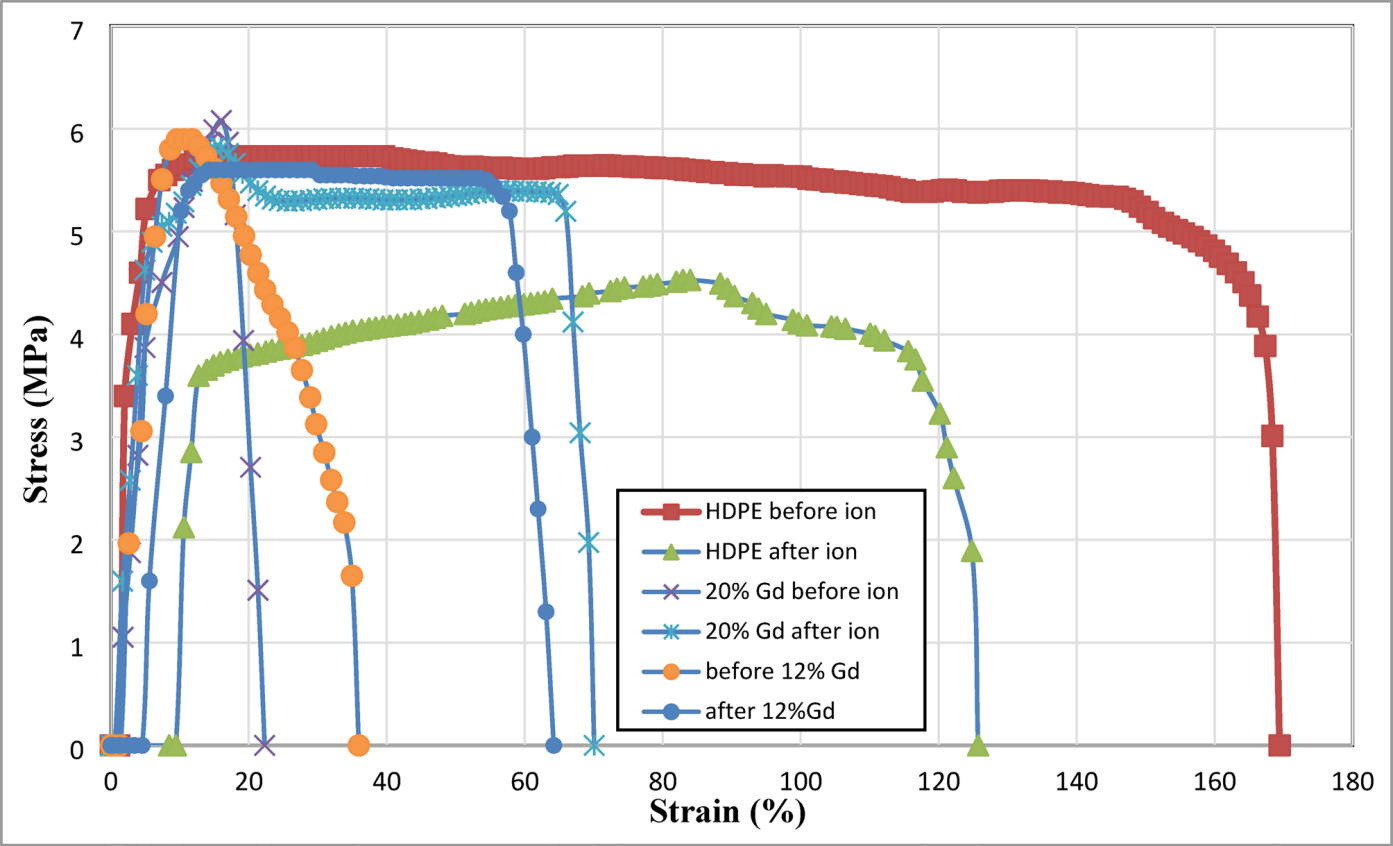
Stress-strain curves with different ratios of Gd_2_O_3_/HDPE composites.

The mechanical properties of 12% and 20% Gd_2_O_3_/HDPE composites before and after ion exposure showed structural changes due to argon ion irradiation, as shown in Table 2; Fig. 9. Young’s modulus, which represents material stiffness, shows decrease in both HDPE and the Gd composites post-ion exposure. HDPE originally exhibits a high modulus of 181 N, indicating strong rigidity, but after irradiation, it drops sharply to 78 N, suggesting a weakening of its structural integrity. while Gd-doped samples also softened, with the 20% Gd sample decreasing from 38 N to 30 N and the 12% Gd sample from 53 N to 41 N. Tensile stress decreased post-irradiation, with HDPE dropping from 5.73 MPa to 4.53 MPa, indicating reduced resistance to applied stress. Interestingly, the 12% Gd sample showed a slight increase in tensile stress (5.6 MPa to 5.92 MPa), which could indicate minor strengthening due to radiation-induced cross-linking effects. The most notable trend appears in elongation at break, where HDPE experienced a decline (168.35% to 124.82%), but Gd-doped samples showed a significant increase (20% Gd: 21.33% to 70.06%; 12% Gd: 35.94% to 64.19%), suggesting enhanced ductility. This improvement in flexibility may result from polymer restructuring, relaxation effects, or cross-linking induced by irradiation. The findings highlight a trade-off between mechanical stiffness and flexibility, with implications for optimizing gamma radiation and neutron shielding—potentially enhancing the material’s energy absorption while maintaining adequate structural integrity.

**Table 2:** Mechanical properties of different ratios of Gd_2_O_3_/HDPE composites before and after argon ion irradiation.

Sample	Young’s modulus (*N*)	Tensile Stress (MPa)	elongation percentage @ break (%)
HDPE before	181	5.73	168.35
HDPE after	78	4.53	124.82
20% Gd before	38	6.08	21.33
20% Gd after	30	5.81	70.06
12% Gd before	53	5.6	35.94
12% Gd after	41	5.92	64.19

### Shielding properties

#### Gamma-ray shielding

**Fig. 10:**
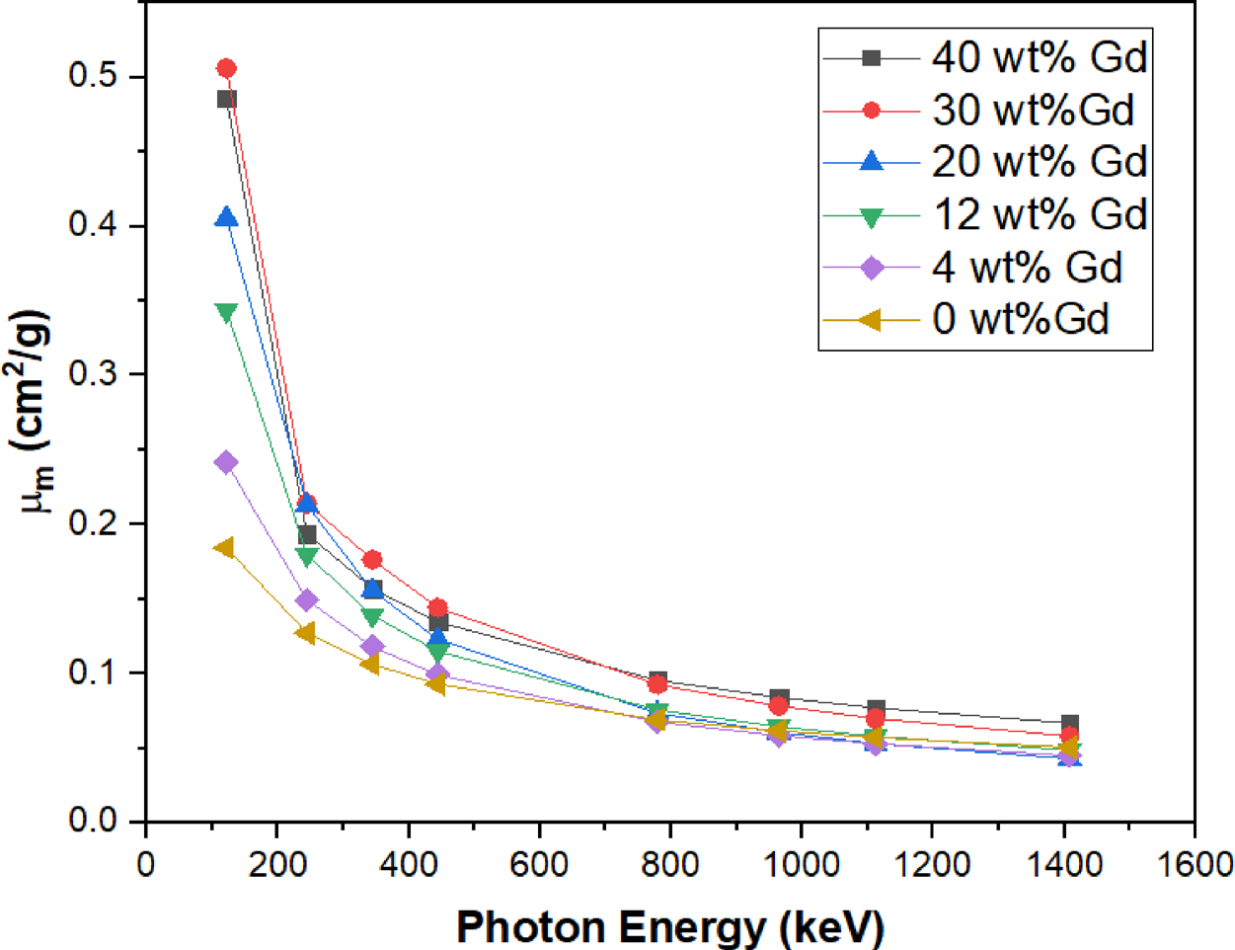
Plotting the µ_m_ experimental data against energy (statistical error less than 2%.).

One important metric for evaluating a material’s gamma-ray shielding efficiency is the mass attenuation coefficient (µ_m_). It measures the likelihood that the material will interact with γ-rays per unit mass, which is a crucial parameter for determining the effectiveness of the shielding. Table 3 summarizes the µₘ of Gd₂O₃/HDPE composites at different filler ratios. The experimental results of µ_m_ are plotted against the amount of Gd_2_O_3_ nanoparticles in the HDPE matrix Fig. 10. It is evident that the µ_m_ rises with increasing Gd_2_O_3_ particle loading at lower energy levels. All samples exhibit the highest µ_m_ values at the lowest energy (121 keV). The photoelectric effect, more noticeable in high atomic number materials like Gd, causes attenuation to be greater at lower energies. As energy increases, Compton scattering dominates, causing attenuation to decrease more gradually. At low photon energies (e.g., 121 keV), where the coefficient increases greatly from 0.185 cm²/g at 0% Gd to 0.486 cm²/g at 40% Gd. Attenuation variation between different Gd concentrations decreases at higher energies (≥ 779 keV), suggesting that pair production influences the interactions.

**Table 3:** Mass Attenuation coefficients (µ_m_) of different ratios of Gd_2_O_3_/HDPE composites.

Energy (KeV)	0 wt%Gd	4 wt% Gd	12 wt% Gd	20 wt% Gd	30 wt%Gd	40 wt% Gd
121	0.1845	0.2417	0.3440	0.4056	0.5065	0.4861
244	0.1274	0.1495	0.1799	0.2132	0.2141	0.1937
344	0.1062	0.1181	0.1392	0.1556	0.1765	0.1572
444	0.0929	0.0992	0.1150	0.1231	0.1443	0.1346
779	0.0690	0.0675	0.0756	0.0735	0.0926	0.0956
964	0.0617	0.0583	0.0645	0.0605	0.0782	0.0840
1112	0.0572	0.0529	0.0579	0.0531	0.0699	0.0770
1408	0.0505	0.0450	0.0486	0.0427	0.0580	0.0667

**Fig. 11:**
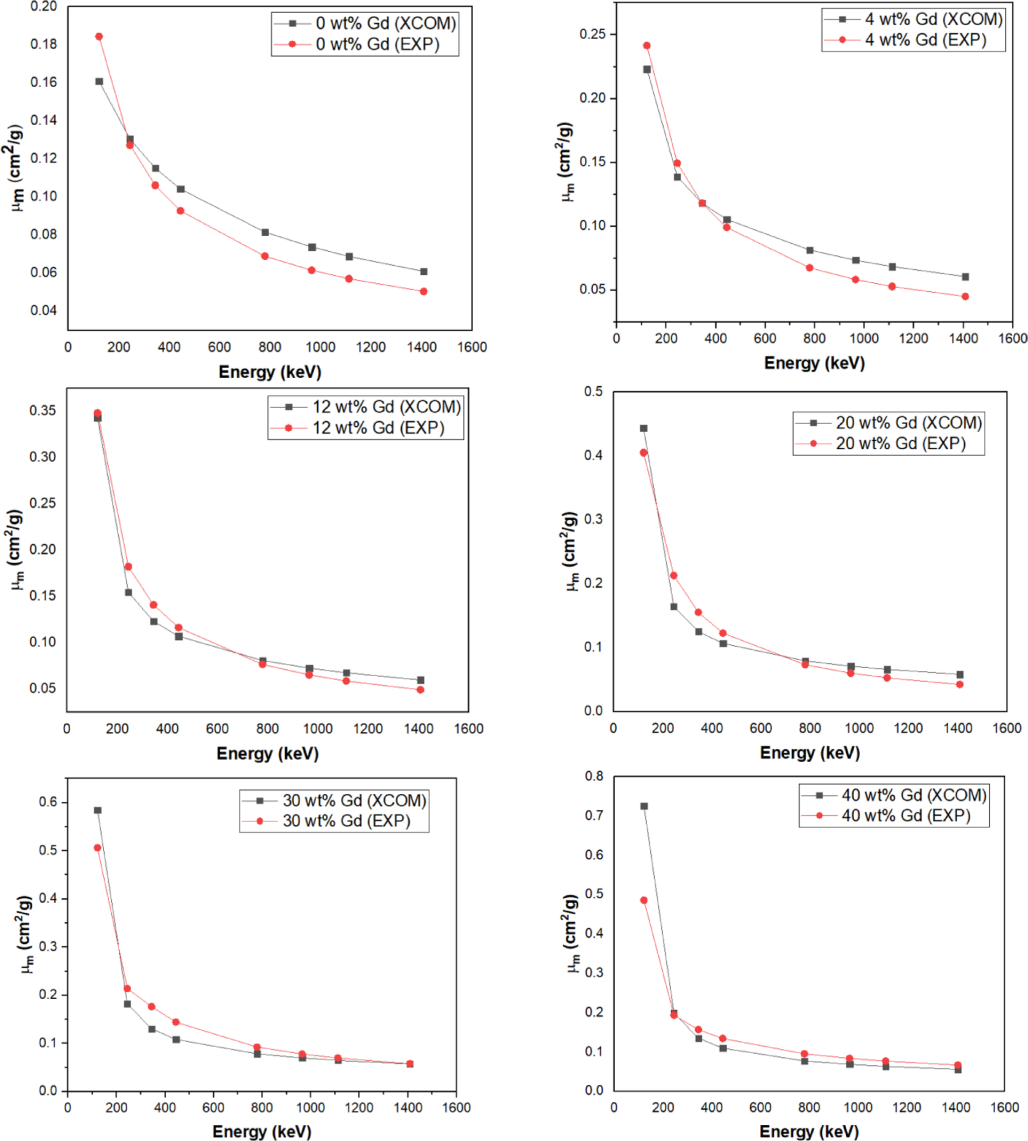
Presents the experimental µₘ values and the corresponding simulation outcomes from XCOM software across several composite materials.

Comparison between the experimentally recorded µ_m_ with the theoretically evaluated Results acquired through XCOM software at the given γ-ray energies for overall various composites, is shown in Fig. 11, the accuracy of the experimental measurements was verified. At the higher loading of 40 wt%, the observed inconsistency is likely due to nanoparticle agglomeration, which leads to uneven distribution and reduces the shielding effectiveness.

It’s clear that irradiation with the argon ion beam makes improvements in the attenuation of γ-rays, especially in the low energy range. It’s probable that the particular sputtering yields under these experimental conditions resulted in a notable relative enrichment of Gd_2_O_3_ at the surface layer, even though preferred sputtering of HDPE was expected. Particularly for low-energy gamma rays, this enriched layer may somewhat improve the overall attenuation if it is uniformly dense. However, because of the shallow implantation of argon ions, this effect was negligible at the higher gamma energies used. This effect may be due to the phonon effect and recoils atom in the target; this leads to increase the crystallite size and breaking some bonds^17^.

**Fig. 12:**
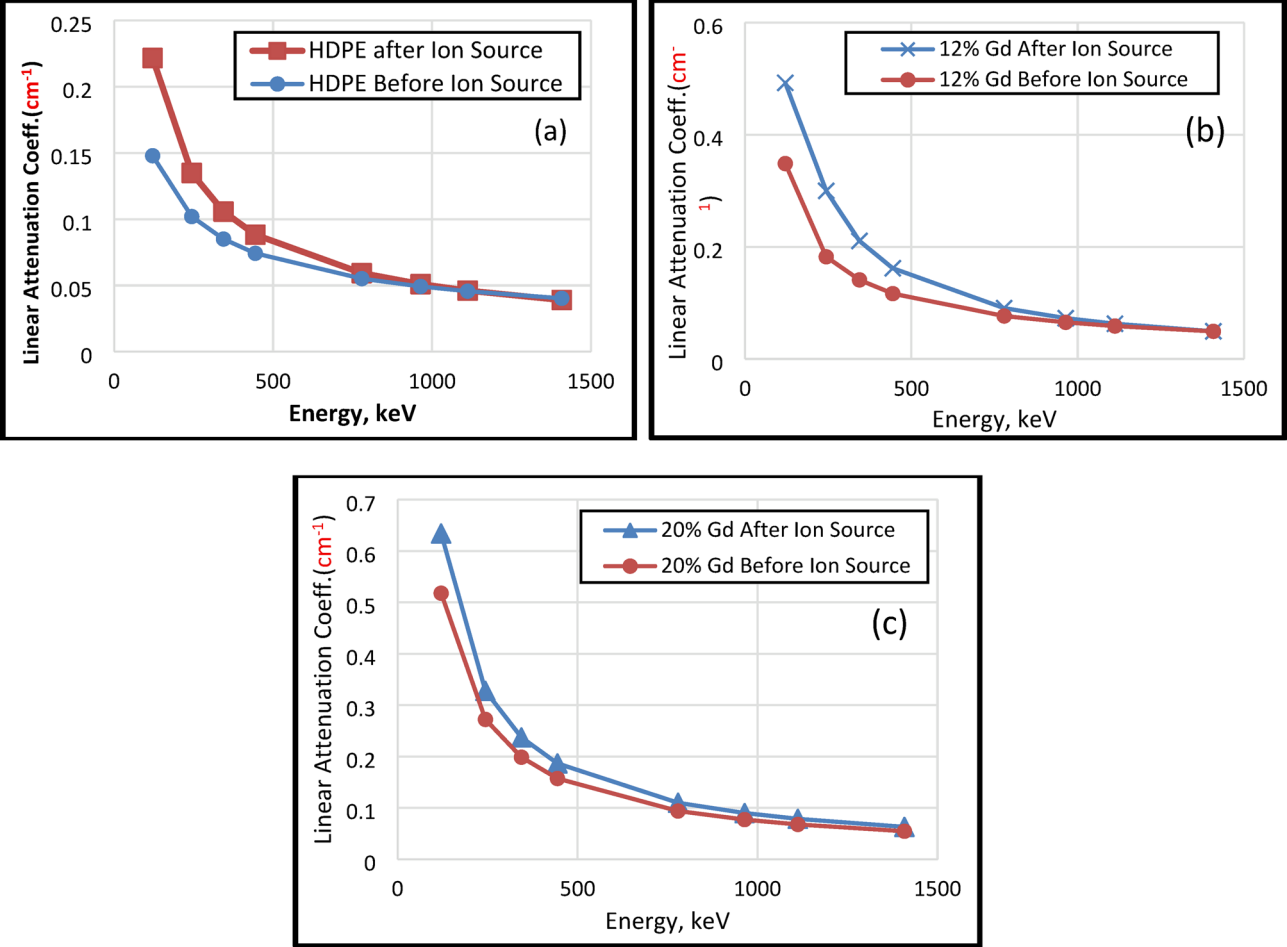
Experimental µ_m_ (Before and after ion beam irradiation) with (**a**) HDPE, (**b**)12.0 wt%, and (**c**) 20.0 wt% Gd_2_O_3_ addition in HDPE matrix.

By comparing the ratios of the linear attenuation coefficient (µ) after ion irradiation to that before, it is possible to quantify the effect of argon ion beam irradiation on the µ of HDPE and Gd_2_O_3_/HDPE composites as illustrated in Fig. 12. Throughout the energy range, the ratios of µ before and after ion irradiation for pure HDPE continuously above 1, signifying an enhancement. A more noticeable effect at lower energies is suggested by this improvement, which varies from roughly 1.50 at 121 keV to 1.14 at 1112 keV and then slightly decreases to 0.97 at 1408 keV. The improvement ratio for the 12% Gd_2_O_3_ composite is even more notable at lower energies, peaking at around 1.41 for 121 keV and progressively dropping to about 1.00 at the highest energy. Likewise, at lower energies, the 20% Gd_2_O_3_ composite shows the greatest increase, with a ratio of roughly 1.22 at 121 keV. From there, it drops to 1.16 until 1112 keV and 1.15 at 1408 keV. For both the pure polymer and the Gd_2_O_3_/HDPE composites, these ratios show that the argon ion beam irradiation has a positive effect on the µ, especially at lower gamma ray energies. This suggests that the interaction probability per unit mass is slightly higher following surface modification.

#### Neutron shielding

**Fig. 13:**
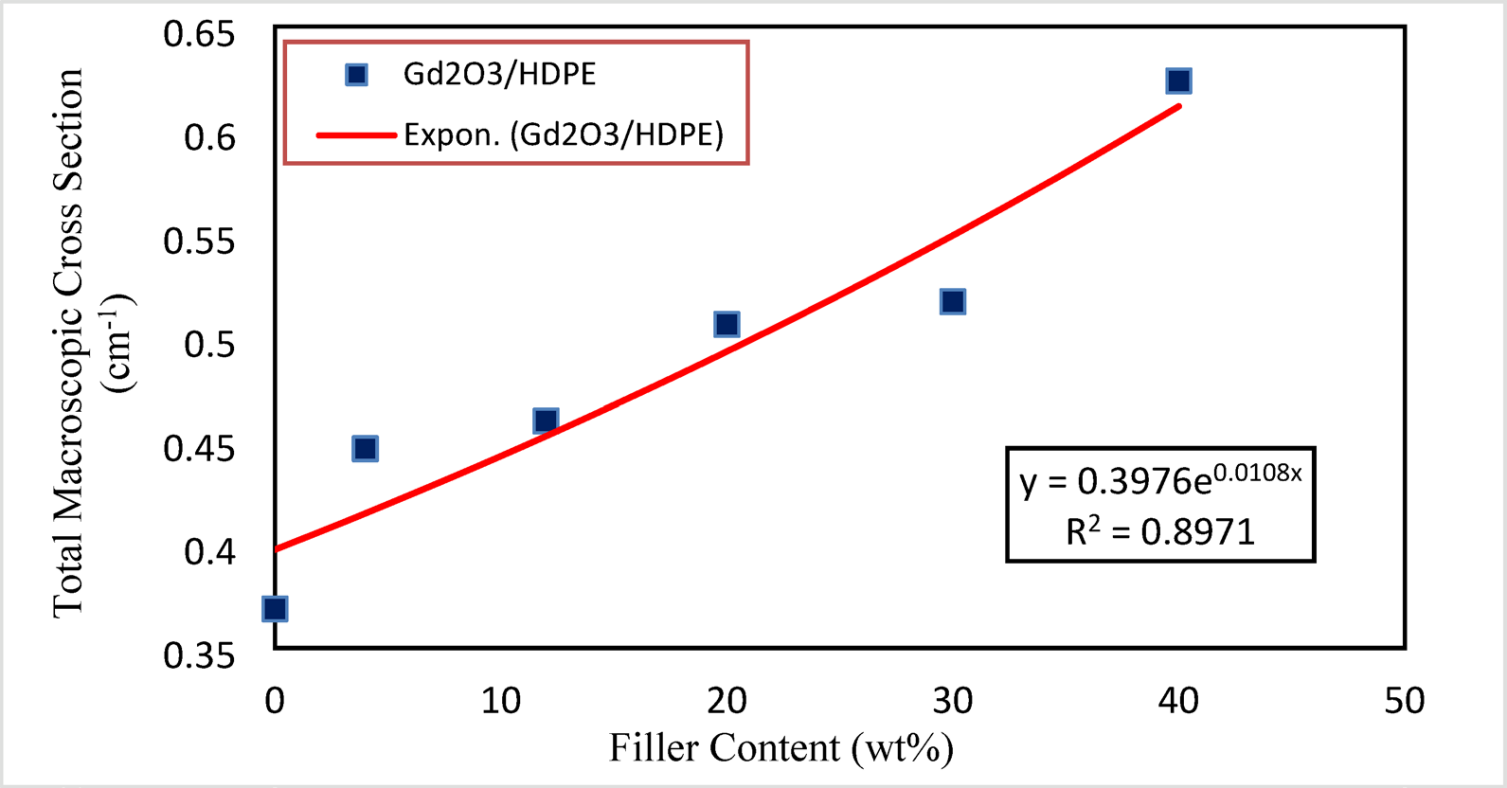
Plot of the total macroscopic cross-section (cm^− 1^) of the assessed Gd_2_O_3_/HDPE nanocomposite samples against the sample content (wt%) (statistical error less than 5%.).

Total macroscopic cross-section (Σ_T_) in units cm^− 1^ is a value explains how likely it is that a neutron will interact with a thick layer of target nano material (Gd_2_O_3_/HDPE). Σ_T_ values for Gd_2_O_3_/HDPE composites show a unique trend as illustrated in Fig. 13. the probability of neutron interaction increases with increasing gadolinium oxide content. This aligns with the well-established act of Gd as a strong neutron absorber, increasing the shielding capacity of the HDPE matrix. The significant increase from (0.369 ± 0.04 cm⁻¹) for 0 wt% to (0.624 ± 0.05 cm⁻¹) for 40 wt% highlights the composite potential for neutron attenuation applications.

**Fig. 14:**
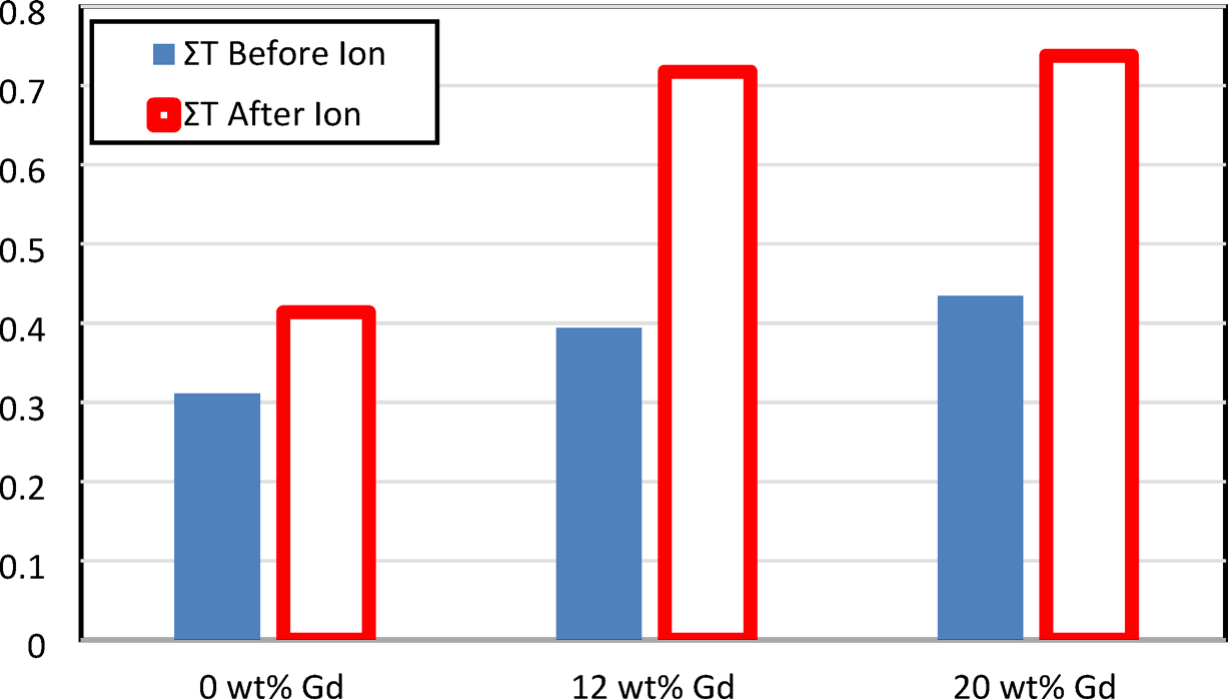
Σ_T_ for the prepared samples before and after argon ion irradiation.

Σ_T_ for Gd_2_O_3_/HDPE composites before and after argon ion irradiation is measured as presented in Fig. 14. The Σ_T_ values for Gd_2_O_3_/HDPE composites explain a remarkable increase following exposure to an argon ion source, indicating that the characteristics of neutron interactions are greatly affected by ion bombardment. Before ion treatment, Σ_T_ values ranged from 0.311 ± 0.03 cm⁻¹ for 0 wt% Gd to 0.434 ± 0.04 cm⁻¹ for 20 wt% Gd, showing the expected increase in neutron absorption with higher gadolinium oxide content because of the high neutron attenuation capacity of Gd. However, after argon ion exposure, Σ_T_ values rise clearly, with the 12 wt% sample increasing from 0.394 ± 0.03 cm⁻¹ to 0.718 ± 0.04 cm⁻¹ and the 20 wt% sample reaching 0.738 ± 0.05 cm⁻¹. These effects are due to increase the vacancies act as neutrons traps, thus improving shielding properties^17^.This suggests that ion bombardment modifies the composite’s atomic or structural characteristics, changes the materials to improve the effectiveness of neutron interactions. Also, the formation of a new nanocrystalline phase is likely attributed to the precipitation effects of an argon ion beam at the grain boundaries, which in turn caused a reduction in the neutron mean free path through the irradiated samples.

## Conclusion

The present work is well-developed and characterized by gamma-ray and neutron shielding composite materials made from HDPE with xGd_2_O_3_ nanoparticles with ratios (x = 4.0%, 12.0%, 20.0%, 30.0%, and 40%) by using sol-gel method. The prepared nanocomposites were characterized by XRD, FTIR, SEM, and EDX techniques. These composites were exposed to argon ion irradiation to enhance their shielding properties. The presence of argon can potentially enhance the dispersion of Gd_2_O_3_-NPs with HDPE matrix, leading to more homogeneous distribution and potentially improving the shielding performance. The mechanical results highlighted a trade-off between mechanical flexibility and stiffness, with indications for optimizing gamma radiation and neutron shielding ability, possibly enhancing the material’s shielding while sustaining suitable structural integrity. The embedded Gd_2_O_3_ significantly improved the attenuation of gamma rays across different energies from 121 to 450 keV. The experimental results demonstrate an acceptable agreement with the theoretical study from XCOM software, supporting the strength of Gd_2_O_3_ in improving shielding achievement. The experimental results indicate that argon ion beam irradiation has a beneficial influence on the µ_m_ for the Gd_2_O_3_/HDPE composites, particularly at lower gamma ray energies. The results show that increasing the ratio of Gd_2_O_3_ in the HDPE matrix remarkably improves the neutron macroscopic cross-section (Ʃ_T_). Starting from 0.369 cm⁻¹ for 0 wt% Gd_2_O_3_, the neutron macroscopic cross-section steadily rises to 0.624 cm⁻¹ for 40 wt% Gd_2_O_3_, reflecting a nearly 69% improvement. Also, the data suggest that exposure to an argon ion irradiation significantly rises the neutron macroscopic cross-section (Ʃ_T_) of Gd_2_O_3_/HDPE composites. For the 0 wt% Gd_2_O_3_ samples, Ʃ_T_ increases by about 33%, while the 12 wt% Gd_2_O_3_ and 20 wt% Gd_2_O_3_ samples show additional improvements of approximately 82% and 70%, respectively. Gd₂O₃/HDPE composites provide effective γ-ray and neutron shielding, making them suitable for radiation protection, especially in personal protective equipment, while maintaining a balance between mechanical flexibility and stiffness for structural integrity and comfort.

## Data Availability

All data generated or analyzed during this study are included in this published article.
